# Lectins from the Red Marine Algal Species *Bryothamnion seaforthii* and *Bryothamnion triquetrum* as Tools to Differentiate Human Colon Carcinoma Cells

**DOI:** 10.1155/2009/862162

**Published:** 2009-12-09

**Authors:** Vicente P. T. Pinto, Henri Debray, Danuta Dus, Edson H. Teixeira, Taianá Maia de Oliveira, Victor Alves Carneiro, Alrieta H. Teixeira, Gerardo C. Filho, Celso S. Nagano, Kyria S. Nascimento, Alexandre H. Sampaio, Benildo S. Cavada

**Affiliations:** ^1^Curso de Medicina/Campus de Sobral, Universidade Federal do Ceará, Fortaleza 60020-181, Brazil; ^2^Laboratoire de Chimie Biologique, UMR N. 8576 CNRS, Université des Sciences et Technologies de Lille, 59655 Lille, France; ^3^Institute of Immunology and Experimental Therapy, Polish Academy of Sciences, 53-114 Wroclaw, Poland; ^4^BioMol-Lab, Departamento de Bioquímica e Biologia Molecular, Universidade Federal do Ceará, Fortaleza 6043, Brazil

## Abstract

The carbohydrate-binding activity of the algal lectins from the closely related red marine algal species Bryothamnion triquetrum (BTL) and Bryothamnion seaforthii (BSL) was used to differentiate human colon carcinoma cell variants with respect to their cell membrane glyco-receptors. These lectins interacted with the cells tested in a dose-dependent manner. Moreover, the fluorescence spectra of both lectins clearly differentiated the cells used as shown by FACS profiles. Furthermore, as observed by confocal microscopy, BTL and BSL bound to cell surface glycoproteins underwent intense internalization, which makes them possible tools in targeting strategies.

## 1. Significance

To the best of our knowledge, this report is the first full investigation demonstrating composition differences in cancer cell membranes detected by agglutinating proteins from marine algae. In addition, according to our results, *Bryothamnion seaforthii *(BSL) and* Bryothamnion triquetrum* (BTL) lectins exhibit distinct profiles for the cell types assayed. These differences could be due to the presence of distinct *glyco-receptors*, different levels of their expression on the cell surface, or even by discrepancy in the affinity profile of these lectins for these receptors.

## 2. Introduction

The carbohydrate-binding activity of lectins has been proven to be responsible in promoting various cells, of different origins, to produce or mediate phenomena as distinct as neutrophil adhesion and rolling during inflammatory processes or nitric oxide induction in macrophages [1, 2]. Numerous lectins have become a powerful tool in the structural characterization of purified or membrane-attached glycoconjugates, as well as in studies on cell behavior [3–5].

The use of lectins in the therapy of many diseases has been discussed elsewhere [6, 7], but this potential is still far from being fully exploited. On the other hand, these proteins have become well-established means for understanding various aspects of cancer and metastasis, given that several studies have shown the modification of surface carbohydrates upon malignant transformation, tumor cell differentiation, and metastasis [8, 9], and lectins are, in several cases, good candidates for detecting such changes.

The applications proposed for lectins in oncology include their use as diagnostic probes [10], biological response modifiers (e.g., immunomodulatory effects of the mistletoe lectin ML-1 which result in dramatic antitumor effect) and as carriers for drug delivery and targeting [6, 11].

Marine algal lectins seem to be especially interesting for biological applications, because they are in general, small molecules (thus, thought to induce minor immunogenicity), possess great stability on account of their several disulfide bridges, and show high specificity for complex carbohydrates and glycoconjugates, such as the ones present on cell surfaces [12–14].

In the present study, we have combined flow cytometry with confocal microscopy to examine glycoconjugates expressed on cancer cell membranes and to discriminate metastatic variants of human colon carcinoma cells, using two marine algal lectins PITC-labeled, purified from the red marine algae *Bryothamnion seaforthii* and* Brythomnion triquetrum*, named BSL and BTL, respectively. Bovine serum albumin (BSA) PITC-labeled was used as negative control in the assays.

## 3. Experimental Procedures

*Lectins*. Purified BSL and BTL were obtained after ion exchange chromatography on a DEAE-cellulose column according to the procedure described in references [15, 16]. The agglutinating fractions of each protein peak of both algae were pooled, dialyzed, and lyophilized. The purity was confirmed by 12.5% SDS-PAGE [17]. PITC-labeling (Molecular Probes, Inc) of the lectins was performed in 2.0 mL of 0.1 M carbonate/bicarbonate buffer, pH 9.3, and ethylene glycol (3 : 1 v/v), using a lectin/PITC ratio of 1 : 100. The mixture was submitted to constant mixing for 5 hours, at 4°C, in the dark. After incubation, the lectin-PITC complex was separated from noncomplexed PITC by molecular exclusion chromatography using a PD10 column (Amersham Bioscience) equilibrated with water containing 5% N-butanol. The fractions containing the labeled-lectins were recovered, dialyzed, and lyophilized.

*Cells*. The human colon adenocarcinoma cell line LS-180 was obtained from Deutsche Krebsforschungzentrum, Heidelberg, Germany. The LS 180 cells originated from a 58-year-old Caucasian female with colon carcinoma Duke's type B. The cells produce a high level of carcinoembryonic antigen (CEA) and express on their surface the tumor-associated carbohydrate antigenic epitopes Lewis X, sialyl Lewis X, and Lewis Y. 

The endothelial cell line HPLNEC.B3—human microvascular endothelial cells from a peripheral lymph node of a patient with Hodgkin's lymphoma—was isolated and characterized, following procedures described in [18]. Cell lines and the further selected variant sublines were grown in OptiMEM medium supplemented with 3% fetal bovine serum (all reagents from Gibco, Grand Island, N.Y.). Cells were cultured in 25-cm^2^ TC flasks at 37°C in a 5% CO_2_-95% humidified air (Falcon or Costar) atmosphere and passaged weekly, using 0.25% trypsin/0.05% EDTA solution (Gibco, Grand Island, N.Y.). 

The in vitro selection of LS180 (EB3) cells with increased affinity for human HPLNEC.B3 microvascular endothelial cells was carried out according to [19]. The EB3 variant was subjected to further selection by inoculation in vivo, in athymic NCr *nu/nu *male mice, by various routes of inoculation (intravenously, intrasplenically, or orthotopically), to select differentially metastasizing variants [20]. After repeated passages, several highly metastatic cells variants were obtained. For the lectin binding experiments, LS 180, EB3, and three variants were chosen: the first variant, LS-180EB3 5W (5W), metastasizes preferentially to the liver, after orthotopic (into intestinal wall) transplantation; the second one, LS-180EB3 3LNLN (3LNLN), metastasizes to peripheral lymph nodes after intravenous inoculation, and the third one, LS-180EB3 8W (8W) variant, forms metastases in the liver, after intrasplenic inoculation. The cells were propagated in OptiMEM medium supplemented with 5% fetal bovine serum and 2 mM glutamine (all reagents from Gibco, Life Sci.) at 37°C, 5% CO_2_-95% humidified atmosphere. The cells were cultured in 25-cm^2^ flasks (Falcon) and later passaged using 0.25% trypsin/0.05% EDTA. Since *Mycoplasma* contamination of cell culture systems causes major problems for basic research, samples of each cell variant were screened for contamination with a *Mycoplasma *detection kit enzyme immunoassay (Boehringer Mannhaeim, Germany). The results were negative (data not shown). 

To choose the lectin concentration to be used in the assays of lectin-cell-interaction, different doses of each lectin were initially evaluated (1, 2, 5, 10, 15, 20, and 25 *μ*g/mL). 

For lectin-cell-interaction analysis, cells were collected using 0.05% EDTA in 10 mM phosphate-buffered saline, pH 7.4 (PBS), and suspended (2 × 10^5^ cells) in 200 *μ*L of PBS containing 0.1% BSA. The cells were incubated with different concentrations of PITC labeled-lectin in 200 *μ*L of PBS/0.1% BSA for 1 hour at 4°C, then centrifuged (1000 × g for 5 min), and afterward washed to eliminate the noninteracting PITC labeled-lectins. The cells were recovered in 1.0 mL PBS and analyzed in a FACSCalibur flow cytometer (Becton-Dickinson). Live cells (5000 counts) were acquired for each data file. The data were processed and the mean fluorescence intensity was calculated by the Cell Quest Software (Becton-Dickinson). The confocal microscopy analysis was performed using aliquots containing 50 000 cells in 250 *μ*L PBS/0.1% BSA to which was added 150 *μ*L of PBS containing lectin (25 *μ*g/mL for BODIPY-lectin binding and 10 *μ*g/mL for PITC-lectin binding). The resultant material was then divided into two glass tubes and incubated, separately, at 4°C and 37°C, for 45 minutes in the dark. After incubation, the suspension was centrifuged (1500 × rpm) for 5 minutes at 4°C. The supernatant was discarded, and the pellet recovered in 100 *μ*L of PBS containing 1% paraformaldehyde; the cells were then incubated for 45 minutes at room temperature. After vortexing the cell suspension, an aliquot of 10 *μ*L was placed on a glass slide and coverslipped. The interaction was analyzed approximately 4 hours later, using a ZEISS Axiovert S 100 microscope, with a confocal system, Bio-Rad MRC 1024, equipped with a 488-nm argon laser.

## 4. Results

**Figure 1:**
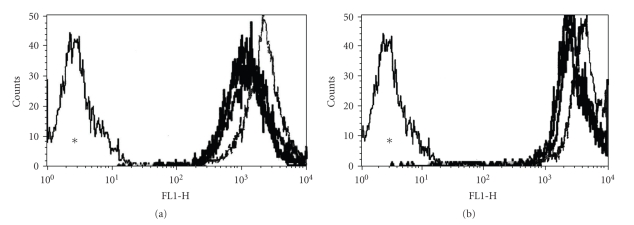
Analysis by flow cytometry of (a) *Bryothamnion seaforthii *lectin, BSL, and (b) *Bryothamnion triquetrum* lectin, BTL PITC, labeled. In this assay lectins were used at 10 *μ*g/mL. (*) negative control BSA PITC-labeled.

The PITC-labeled algal lectins BSL and BTL were allowed to interact with LS180, EB3, and three colon carcinoma cell variants (3LNLN, 5W, and 8W). Analysis by flow cytometry demonstrated that both PITC-labelled proteins were able to recognize cell membrane components in these cells (Table 1). The recognition of cell surface components indicates that these lectins were able to differentiate cancer cell variants (Figure 1). The shift in fluorescence intensity induced by the interaction of the PITC-lectins with the cell variants was quite similar, suggesting that the lectins bound to the cell surface may be anchored by the same component. It is interesting, however, that both lectins could differentiate the cells tested under the same experimental conditions. Analysis of the interaction, with regard to cell recognition, showed that BSL and BTL discriminated the cell variants.

**Figure 2:**
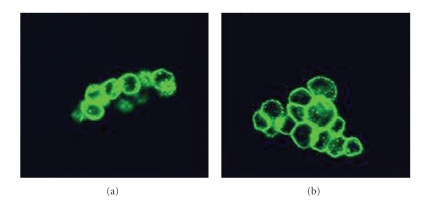
Confocal microscopy showing the interaction of (a) *Bryothamnion seaforthii* lectin, BSL, and (b) *Bryothamnion triquetrum* lectin, BTL PITC, labeled (at 4°C for 1 hour) with EB3 colon carcinoma cells variant. In this assay lectins were used at 10 *μ*g/mL. In each case the lectin uptake was weakly visualized.

**Figure 3:**
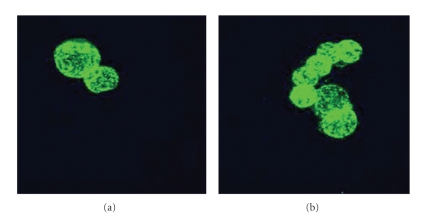
Confocal microscopy showing the interaction of (a) *Bryothamnion seaforthii* lectin, BSL, and (b) *Bryothamnion triquetrum* lectin, BTL PITC, labeled (at 37°C for 1 hour) with EB3 colon carcinoma cells variant. In this assay lectins were used at 10 *μ*g/mL. In each case the lectin uptake was strongly visualized.

Temperature variables (4°C and 37°C) were used to verify differences in the binding properties of PITC-lectins. This step was necessary since the binding of the lectin to the membrane elements is very likely to take place at 4°C, where the fluidity of the cell membrane is reduced and the energy consumed in the transport processes is limited. At 37°C, a strong interaction of the lectins with the glycocalix components usually induces their uptake and storage in acidic vacuoles such as the lysosomes [21]. The binding of the lectins observed by flow cytometry was confirmed by confocal analysis at 4°C and no differences in the labeling-patterns of BSL and BTL could be observed (Figure 2). Nonetheless, when the bioadhesive activity was evaluated at 37°C, some particularities were observed. In general, internalization was quite evident (Figure 3). 

## 5. Discussion

The haemagglutinating activity in extracts of the red marine algae *B.seaforthii *and *B. triquetrum *was first reported by our group [22, 23]. Their agglutinating fractions mainly recognized rabbit, chicken, and cow trypsin-treated erythrocytes. Purified BTL is an 8.9-kDa cysteine-rich protein [16] and mono- di- and trisaccharides do not inhibit its haemagglutinating activity [15]. This latter property together with the amino acid sequence [16] segregates these proteins from the other groups of plant lectins, which are naturally grouped on the basis of their monosaccharide specificity or sequence homology. Since these proteins seem to possess carbohydrate-binding specificity distinct from well-known plant lectins, they have emerged as a new tool to test different activities already investigated using plant lectins, especially in the characterization of glycoconjugates. 

According to our results, the two lectins exhibit distinct profiles for the cell types examined. These differences could be caused by the presence of distinct glycoreceptors, different levels of their expression on the cell surface, or still by a discrepancy in the affinity profile of these lectins in relation to these receptors. On the other hand, no significant interaction was observed among the negative control (BSA) and cells. BSA was added because it is a protein with well-known physicochemical characteristics, without lectin activity and that it is able of binding with PITC. 

Unusual glycosylation of the cell surface proteins has been frequently associated with the characteristics of malignant cells. These include increased expression of Lewis-related antigens, polylactosamine, and *β*-(1→6)GlcNAc branching at the trimannoside core of the *N*-linked oligosaccharides and structures of mucin-type *O*-linked glycans [24–26]. Evidence has accumulated that some of these abnormal glycosylations are important for tumor progression and development of distant metastasis [27]. 

Abnormal glycosylations were also observed in mucins. They are complex, carbohydrate-rich O-type glycoproteins secreted by mucosal and submucosal glands and are thought to act primarily as a physical/chemical barrier to pathogens and particles [28, 29]. Mucins can be broadly classified as either secreted or transmembrane. Differential expression of these glycoproteins has been correlated with malignant transformation [30]. In fact, the expression of many cell surface mucins is perturbed in colorectal neoplasia. MUC1 and MUC13, for instance, are highly expressed in such carcinomas when compared to normal colon cells [17, 29].

It is well known that the glycoproteins mucins are strong specific inhibitors of the hemaglutinating activity of lectins, in particular those isolated from marine algae [12–15]. In fact, mucin has been used as affinity chromatography matrix for lectin purification [31], as well as inhibitor for biological functions expressed by marine algae lectins [32–34].

Given the ability of both lectins presented here to bind to mucins and based on the aforementioned data and on ours, we speculate that one or both of these glycoproteins may be the receptors recognized by BSL and BTL on the carcinoma cells tested here. 

The carbohydrate-binding activity of lectins has been very useful in studying alterations in cell membrane glycosylation, although the complexity of this problem could not be tackled by a single approach. In fact, lectin binding to carbohydrate-derivative self-assembled monolayers, showed that the same lectins may switch from one carbohydrate ligand to another as the surface density of the carbohydrate-ligands increases [35]. This fact exposes the dimension and complexity of the problem and suggests that to study or map natural or aberrant cell surface glyco-receptors, not one but a set of well-characterized lectins with similar ligand capacity, should be available. 

It was observed that for both lectins the interactions with different cells increased proportionally with the different concentrations tested, until 10 *μ*g/mL. Above this concentration (10 mg/mL), it can have saturation of the glycoconjugates recognized by the lectins. Thus, the interaction would not increase when larger concentrations of lectins are used. According to our results, BSL and BTL could be exploited in investigating structural modification of cell membrane glycoconjugates on cell systems as we have tested in the present study. However, more detailed investigations into their precise glycan-binding specificity should be carried out. 

The difference in profiles shown by the highly similar lectins BSL and BTL for the cell types studied is rather common for legume lectins, which may show distinct biological activities despite their high similarity in both primary sequence and overall three-dimensional structure [36]. In fact, a previous study from our group has reported very significant differences in the ability of BSL and BTL lectins to prevent streptococci adherence to acquired enamel pellicle in vitro [33]. In addition, unpublished studies of fine specificity conducted by our group, show that BSL and BTL actually exhibit distinct specificities for most glycoconjugates (e.g., bovine submaxillary mucin and porcine gastric mucin) that were tested. Thus, we suggest that these punctual differences in sequence reflect local alterations in the three-dimensional structures. Such alterations are enough to cause a variable carbohydrate affinity, and may constitute the main cause for the distinct profile shown by BSL and BTL. Nevertheless, this speculation hinges on the fact that the lectin-receptor interaction is a protein-carbohydrate interaction, which could not be demonstrated by inhibition assays in the lectin-cell interaction system using classical plant lectin-inhibitory saccharides, such as galactose, glucose, *α*-methylmannoside, or lactose, as expected, given that there are no studies to date reporting an algal lectin whose specificity is directed toward mono- or disaccharides.

The biological signal transduction properties of lectins, in general, appear to be due to their ability to bind and then cross-link glycan receptors. This cross-linking leads to changes in the interaction between receptor and cytoskeletal proteins and concomitant alterations in the mobility and aggregation of other surface events, including the ones responsible for the internalization of BSL and BTL.

The finding that the binding of the lectins to the carcinoma cells and the observation of its internalization are quite appealing since they could be used as carriers in cancer-targeted therapy [6]. There is evidence that drugs that bind better to cancer cells than to normal ones are internalized [37], but if this drug is bound to a lectin which is internalized, not only the specificity but the potency of the drug could be enhanced. 

In summary, we demonstrated that the algal lectins BSL and BTL were capable of differentiating human colon carcinoma cell variants with respect to their cell membrane glyco-receptors and could be exploited to investigate structural modification of cell membrane glycoconjugates in cancer cell systems. In addition, we showed that the binding of both lectins to the carcinoma cells results in their internalization, which is a very interesting property that could be used in future applications, such as drug delivery.

## Figures and Tables

**Table 1:** Interaction of PITC-lectin complexes (expressed in fluorescence units, FU*) with human colon cancer cells.

Cell variant	PITC-BSA** (10 *μ*g/mL)	PITC-BTL*** (10 *μ*g/mL)	PITC-BSL*** (10 *μ*g/mL)
LS 180	18.4 ± 0.9	6397.4 ± 287.8	5839.1 ± 280.3
EB3	21.1 ± 1.05	6904.7 ± 345.2	6599.3 ± 283.7
3LNLN	15.1 ± 0.75	3831.9 ± 180.1	4319.8 ± 215.9
5W	19.3 ± 0.96	3490.2 ± 143.1	3079.4 ± 141.6
8W	12.5 ± 0.68	6139.1 ± 288.5	5484.2 ± 213.9

*Fluorescence detected by FACSort for a total of 5000 events (mean ± s.d.), corrected using standardization with Immuno-Brite (GIBCO).

**BSA-PITC was used as negative control.

*** *P* < .05.
